# Palmitoylated small GTPase ARL15 is translocated within Golgi network during adipogenesis

**DOI:** 10.1242/bio.058420

**Published:** 2021-12-13

**Authors:** Yixing Wu, Ying Bai, David G. McEwan, Liz Bentley, Dimitra Aravani, Roger D. Cox

**Affiliations:** 1 Mammalian Genetics Unit, MRC Harwell Institute, Harwell Oxford, Oxfordshire, OX11 0RD, UK; 2 Division of Cell Signalling & Immunology, School of Life Sciences, University of Dundee, Dundee, DD1 5EH, UK; 3 Tumour Cell Death Laboratory, Cancer Research UK Beatson Institute, Garscube Estate, Switchback Road, Glasgow, G61 1BD, UK

**Keywords:** ARL15, Adipogenesis, Palmitoylation

## Abstract

The small GTPase ARF family member *ARL15* gene locus is associated in population studies with increased risk of type 2 diabetes, lower adiponectin and higher fasting insulin levels. Previously, loss of ARL15 was shown to reduce insulin secretion in a human β-cell line and loss-of-function mutations are found in some lipodystrophy patients. We set out to understand the role of ARL15 in adipogenesis and showed that endogenous ARL15 palmitoylated and localised in the Golgi of mouse liver. Adipocyte overexpression of palmitoylation-deficient ARL15 resulted in redistribution to the cytoplasm and a mild reduction in expression of some adipogenesis-related genes. Further investigation of the localisation of ARL15 during differentiation of a human white adipocyte cell line showed that ARL15 was predominantly co-localised with a marker of the cis face of Golgi at the preadipocyte stage and then translocated to other Golgi compartments after differentiation was induced. Finally, co-immunoprecipitation and mass spectrometry identified potential interacting partners of ARL15, including the ER-localised protein ARL6IP5. Together, these results suggest a palmitoylation dependent trafficking-related role of ARL15 as a regulator of adipocyte differentiation via ARL6IP5 interaction.

This article has an associated First Person interview with the first author of the paper.

## Abstract (precis)

**Summary:** ARL15 (GTPase ARF family) is associated with adipose traits. ARL15 is palmitoylated, localised to Golgi in preadipocytes and translocated to other Golgi compartments during differentiation. ARL15 interacts with ER-localised ARL6IP5.

## INTRODUCTION

The World Health Organisation (WHO) estimates there were 422 million people with diabetes in 2014. Approximately 90% of people with diabetes have Type 2 diabetes (T2D), which is characterised by defects in insulin secretion and insulin action. Increased body mass index (BMI), leading to insulin resistance, is one of the key determinants of lifetime T2D risk (Hippisley-Cox et al., 2009; Narayan et al., 2007). Genome wide association population studies have identified over 243 genetic loci with 403 distinct association signals for T2D (Mahajan et al., 2018). Although many of these loci indicate a role for islet beta-cell function in T2D risk, 26 signals were attenuated by BMI adjustment suggesting T2D risk was driven by adiposity. A further 15 loci were more strongly associated with BMI-adjusted signals with effects on insulin secretion and/or ectopic fat storage (Mahajan et al., 2018). Importantly, there is also genetic evidence of insulin resistance in T2D that is linked to ectopic fat distribution and reduced subcutaneous adiposity (Scott et al., 2014; Yaghootkar et al., 2014). In addition, there are rare monogenic conditions such as lipodystrophy that result in partial or complete loss of adipose tissue and cause severe insulin resistance and diabetes (Semple et al., 2011).

The small GTPase adenosine diphosphate (ADP)-ribosylation factor-like protein 15 (ARL15) gene locus is associated with several metabolic traits including increased risk of T2D, lower adiponectin and higher fasting insulin levels (Richards et al., 2009). This gene locus was also associated with insulin resistance and adiposity (Scott et al., 2014, 2012; Yaghootkar et al., 2014). Further, loss-of-function mutations in the *ARL15* gene have also been identified in lipodystrophy patients (Rocha et al., 2017). These associations suggest a role for ARL15 in adipose tissue homeostasis. Indeed, it has been shown that reduction of *Arl15* impaired adipogenesis in 3T3-L1 pre-adipocytes and reduced adiponectin secretion from mature adipocytes (Rocha et al., 2017). Additionally, it has been shown that siRNA-mediated *ARL15* depletion in a human β cell line (EndoC-βH1) reduces insulin secretion, further linking this gene to diabetes traits (Thomsen et al., 2016). However, the mechanisms by which ARL15 potentially regulate these processes remain unknown.

The family of ADP-ribosylation factor-like proteins (ARL) belong to the small GTPases RAS superfamily that exhibit structural homologies such as the inter-switch toggle and that switch between GTP-bound active and GDP-bound inactive conformations (D'Souza-Schorey and Chavrier, 2006; Kahn et al., 2006). ARF family proteins are largely involved in membrane trafficking and membrane-associated metabolic regulation (Burd et al., 2004; D'Souza-Schorey and Chavrier, 2006; Nie et al., 2003) and ARL family proteins have more diverse subcellular localisations and functions (Burd et al., 2004). For example, activated ARL1 and ARL3 are localised at the trans-Golgi network (TGN) and regulate Golgi trafficking pathways by recruiting Golgi targeting proteins such as the GRIP domain (Panic et al., 2003; Setty et al., 2003); whereas centrosome localised ARL2 and ARL3 play roles in regulating cell morphology and cell cycle by manipulating microtubule-related pathways (Zhou et al., 2006a). In cilia, ARL13B is likely to regulate post-translational modification of tubulin (Larkins et al., 2011). Adipogenesis is a well-regulated multi-step process that is comprised of cell growth arrest, transcriptional activation, morphological changes and Golgi-mediated membrane vesicle trafficking (Tang and Lane, 2012). In addition to showing that ARL15 is an adipogenic regulator (Rocha et al., 2017), overexpression of ARL4D another family member, in 3T3-L1 cells reduced expression levels of several important adipogenesis-related genes such as aP2 (FABP4), fatty acid synthase (FASN), lipoprotein lipase (LPL) and hormone-sensitive lipase (HSL) (Yu et al., 2011), indicating a negative regulatory role of ARL4D on adipogenesis.

In this study, in order to investigate the mechanistic role of ARL15 in regulating adipocyte differentiation, we first investigated endogenous subcellular localisation, post-translational modification and localisation change of ARL15 during adipogenesis. Secondly, we explored potential protein–protein interacting partners of ARL15. These results provide useful information on ARL15 during adipogenic differentiation.

## RESULTS

### Endogenous ARL15 is predominantly localised in the Golgi network

**Fig. 1.**
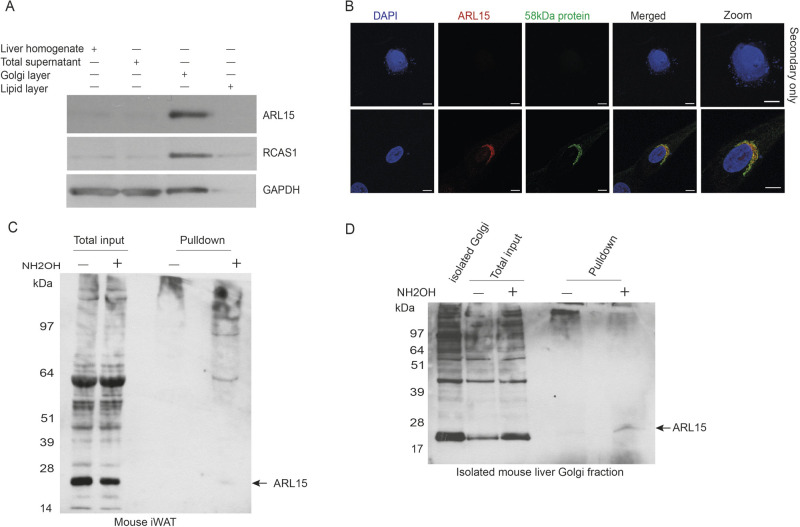
**Endogenous and palmitoylated ARL15 is predominantly localised in Golgi.** (A) ARL15 is found to be enriched in the Golgi fraction. RCAS1: Golgi marker, GAPDH: loading control. (B) Subcellular localisation by immunofluorescence for ARL15. Human white adipocytes co-stained with anti-58 kDa protein. Secondary antibodies Alexa-488 and Alexa-568 conjugated. Scale bars: 10 µm. (C) Detection of ARL15 palmitoylation in purified Golgi of mouse inguinal white adipose tissue (iWAT) by S-acylated resin-assisted capture (RAC) and immunoblotting with anti-ARL15. Total input and eluted fraction from NaCl-containing control (indicated as ‘−’) and NH_2_OH-treated samples (indicated as ‘+’). (D) Palmitoylated ARL15 is present in purified Golgi of mouse liver as detected by RAC and western blot.

Using a protein over-expression method, the localisation of GFP-tagged ARL15 in 3T3-L1 preadipocytes has been shown in the Golgi network (Rocha et al., 2017); whereas in C2C12 myotubes, ARL15 was found to be in the cytoplasm and moved to the perinuclear Golgi region upon insulin stimulation (Zhao et al., 2017). Therefore, we first sought to confirm the localisation of endogenous ARL15 in a purified Golgi network fraction isolated from mouse liver. Western blotting showed that, at steady state, ARL15 was enriched in the Golgi fraction together with a known Golgi marker RCAS1 (Fig. 1A) but not in the visible lipid fraction. We then conducted immunostaining in human white adipocyte tissue derived cell line (hWAT) preadipocytes to confirm the localisation of endogenous ARL15. Immunostaining showed the co-localisation of ARL15 with 58 kDa protein, a known Golgi membrane-associated protein (Bloom and Brashear, 1989; Gao et al., 1998), in the perinuclear region (Fig. 1B). Together, these results clearly demonstrate that endogenous ARL15 is predominantly localised in Golgi.

### Endogenous palmitoylated ARL15 is localised in the Golgi network

Palmitoylation is an important form of post-translational modification (PTM) that regulates protein subcellular localisation, endoplasmic reticulum (ER)-Golgi trafficking and protein–protein interaction (Aicart-Ramos et al., 2011). ARL15 has been identified as a putative palmitoylated protein by mass spectrometry from a human T lymphocyte cell line (Jurkat T cells) (Martin and Cravatt, 2009), mouse adipose tissue and 3T3-L1 adipocytes (Ren et al., 2013). To validate this, a resin-assisted capture (RAC) assay was conducted to first pull down a known palmitoylated protein ARL13B in mouse inguinal white adipose tissue (iWAT). Palmitoylated ARL13B was detectable in the pulldown sample by labelling with anti-ARL13B antibody (Fig. S1). In agreement with the mass spectrometry findings (Ren et al., 2013), palmitoylated ARL15 was detectable in the pulldown sample by labelling with anti-ARL15 antibody (Fig. 1C).

Several important palmitoyl transferases that regulate the palmitoylation process have been found in Golgi (Aicart-Ramos et al., 2011) and ARL15 had been found to be predominantly localised in Golgi. To examine if palmitoylated ARL15 is localised in Golgi, the RAC assay was carried out in the Golgi fraction isolated from mouse liver, where ARL15 is also strongly expressed. Indeed, palmitoylated ARL15 was detectable in the pulldown sample from Golgi fraction by labelling with anti-ARL15 antibody (Fig. 1D).

### Palmitoylation is required for ARL15 to establish its predominant Golgi network localisation

In addition to palmitoylation, prenylation and myristoylation modifications mediate subcellular localisation, protein–protein or protein–membrane interactions (Aicart-Ramos et al., 2011). However, whether ARL15 is prenylated or myristoylated remains unclear. To explore the possibility of ARL15 prenylation, the amino acid sequence of mouse ARL15 was analysed by Prenylation Prediction Suite (PrePS: http://mendel.imp.ac.at/sat/PrePS/index.html). ARL15 neither has a cysteine on the fourth last position as part of a C-terminal CaaX box nor a cysteine within the last five positions of its C-terminal end and is therefore unlikely to be subject to prenylation processes including CaaX Farnesylation, CaaX Geranylgeranylation or Rab Geranylgeranylation. To predict ARL15 myristoylation, the amino acid sequence of mouse ARL15 was analysed by Myristoylator from ExPASy Bioinformatics Resource Portal of Swiss Institute of Bioinformatics (Artimo et al., 2012). Due to a lack of a glycine residue at its N-terminal end, ARL15 was predicted not to be myristoylated.

**Fig. 2.**
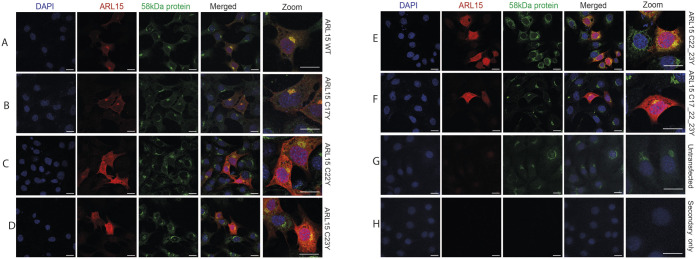
**Palmitoylation is required for ARL15 to establish its predominant Golgi localisation.** Immunostaining against Myc-tag and 58 kDa protein in 3T3-L1 cells overexpressing (A) C-terminal Myc-tagged WT ARL15 and palmitoylation-defect mutants, (B) ARL15 C17Y, (C) ARL15 C22Y, (D) ARL15 C23Y, (E) ARL15 C22_23Y, (F) ARL15 C17_22_23Y, (G) untransfected and (H) secondary antibody only. Secondary antibodies: Alexa-488 and Alexa-568 conjugated. Scale bars: 10 µm.

ARL15 has three palmitoylation sites at its N-terminal (Cys17, Cys22 and Cys23) predicted by a clustering and scoring strategy algorithm CSS-Palm 4.0 (Table S1) (Xue et al., 2011; Zhou et al., 2006b). To test if these cysteine residues are important for the Golgi localisation of ARL15, the three cysteine (C) residues were mutated to tyrosine (Y) through site-directed mutagenesis. The C-terminal Myc tagged wild-type (WT) or N-terminal mutant *Arl15* constructs (C17Y, C22Y, C23Y, C22Y/C23Y and C17Y/C22Y/C23Y) were over-expressed in 3T3-L1 preadipocytes. Subcellular localisation of WT and mutant ARL15 was studied using immunostaining and confocal microscopy. Consistent with endogenous ARL15, over-expressed WT ARL15 was predominantly if not exclusively localised in Golgi (Fig. 2A) (Rocha et al., 2017). Similarly, the C17Y mutant was also localised in Golgi (Fig. 2B) with some plasma membrane accumulation. In contrast to the predominant Golgi localisation of ARL15 the C22Y, C23Y, C22Y/C23Y and C17Y/C22Y/C23Y mutants, each exhibited a cytoplasmic diffused pattern (Fig. 2C–H).

**Fig. 3.**
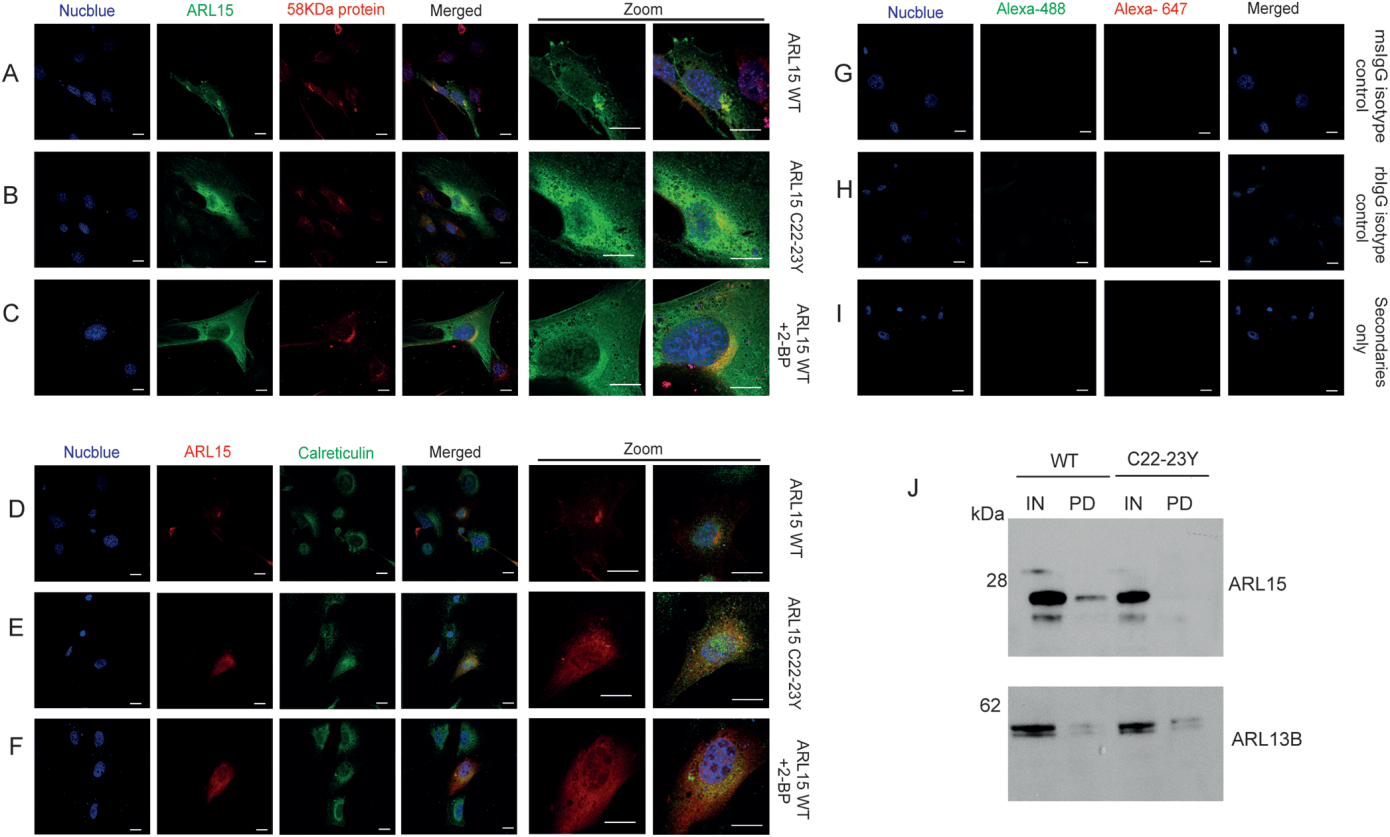
**Residues Cys22 and Cys23 are the palmitoylation sites for ARL15 and are essential for the protein's Golgi localisation.** Immunostaining against Myc-tag and 58 kDa protein in 3T3-L1 cells overexpressing (A) C-terminal Myc-tagged WT ARL15, (B) palmitoylation-defect mutant ARL15 C22_23Y and (C) ARL15 WT treated with 100 µM 2-BP inhibitor. Cells were also co-stained with Myc-tag and Calreticulin (D–F). Species-specific isotype controls and secondary only stained cells shown in G–I. Secondary antibodies: Alexa-488 and Alexa-647 conjugated. Scale bars: 20 µm. (J) Representative image of Acyl-RAC assay followed by immunoblotting against ARL15 and ARL13B in lysates of 3T3-L1 cells expressing Myc-tagged WT ARL15 or ARL15 C22_23Y. Three separate repeats at different passage were performed. IN, input lysates; PD, pull-down lysates.

To further prove that mutant ARL15 disrupted Golgi localisation is due to the palmitoylation defect per se and not due to effects such as misfolding or abnormal degradation of the mutant protein we treated 3T3-L1 cells with 2-bromopalmitate (2-BP), a non-metabolizable palmitate analogue that blocks palmitate incorporation onto proteins. To validate that 2-BP sufficiently inhibits palmitoylation of ARL15, we performed an S-palmitoylation acyl-RAC assay. In cells treated with 100 µM 2-BP overnight, ARL15 was detected in the input fraction and significantly reduced in the pull-down fraction indicating inhibition of palmitoylation compared to the non-treated control cells (Fig. S2A,B). The same was observed for ARL13B, a known palmitoylated protein included as a positive control (Fig. S2C). In order to test the effect of 2-BP inhibition of palmitoylation on ARL15 localisation we transfected 3T3-L1 cells with the C-terminal Myc tagged WT or the C-terminal mutant *Arl15* construct C22Y/C23Y and then treated the WT with or without 2-BP. Immunofluorescence analysis revealed that WT ARL15 expressing cells treated with 2-BP and mutant C22Y/C23Y expressing cells comprised a cytoplasmic and non-specific Golgi (58KDa protein cis-Golgi marker) localisation (Fig. 3A–C,G–I). Co-staining with calreticulin an ER marker revealed again that WT ARL15 is not ER exclusively localised but rather localised in a distinct region reminiscent of Golgi (Fig. 3D). In contrast, WT ARL15 expressing cells treated with 2-BP and cells expressing C22Y/C23Y lost this distinct localisation and appeared to correlate more with ER (Fig. 3D–I). These immunostaining data indicated that residues Cys22 and Cys23 are essential for ARL15 Golgi localisation. However, they do not directly show that these residues are the palmitoylation sites. In order to prove show this and validate the predictive algorithm for residues Cys22 and Cys23 we performed an S-palmitoylation acyl-RAC assay in 3T3-L1 cells expressing either the WT ARL15 protein or the mutant C22_C23 protein. WT ARL15 was detected both in the input and the pull-down fraction whereas C22Y/C23Y mutant protein was only present in the input fraction, indicating loss of palmitoylation in these residues unlike ARL13B which was detected in the pull-down fraction of the mutant protein (Fig. 3J). Taken together, these findings show that Cys22 and Cys23 are the sites of palmitoylation and that this post translational modification is important for ARL15's Golgi localisation.

### ARL15 translocates away from Golgi network membranes during adipogenesis

**Fig. 4.**
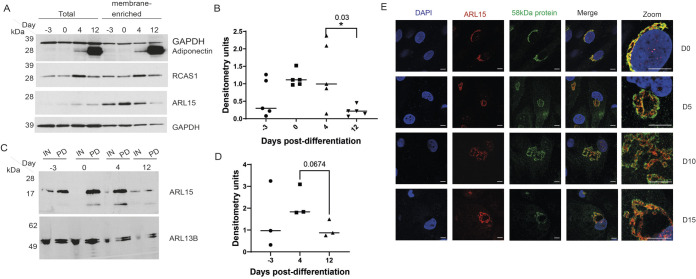
**ARL15 travels within Golgi from the Cis-side to other parts of Golgi during adipogenesis.** (A) Western blot showing ARL15, adiponectin and RCAS1 levels in total cell lysate and membrane-enriched fractions in a time course experiment (days post-differentiation induction). GAPDH and RCAS1 are included for comparison. (B) Level of ARL15 at D12 post-differentiation induction is significantly reduced comparing to D4, when differentiation was induced. ARL15 band intensity is relative to GAPDH. Data are presented as mean±s.e.m. and analysed by using one-way ANOVA. *n*=5 replica experiments; *P<0.05 (C) ARL15 palmitoylation over 12 days of differentiation in 3T3-L1 cells as detected by S-acylated resin-assisted capture followed by immunoblotting with anti-ARL15 and anti-ARL13B. (D) There is a trend towards reduction in ARL15 palmitoylation 12 days post-differentiation. Densitometry is relative to input. Data are presented as mean±s.e.m., analysed by *t*-test between day 4 and day12, *n*=3 repeats with the same cell line. IN, input fraction; PD, pull-down fraction. (E) Representative confocal images of hWAT cells co-stained with anti-ARL15 and anti-58kD protein (Cis Golgi marker). Secondary antibodies: Alexa-488 and Alexa-647 conjugated. Scale bars: 10 µm; Scale bars for zoomed-in image are 5 µm.

Since palmitoylation is a dynamic and reversible membrane association process (Conibear and Davis, 2010), it is probable that ARL15 is not permanently restricted to membrane structures. To study whether the association between ARL15 and intracellular membrane changes during adipogenesis, total 3T3-L1 cell lysate and membrane-enriched fractions from pre-adipogenic (D-3, D0) and post-adipogenic (D4, D12) stages (D0: differentiation induction) were subjected to western blot analysis. Adiponectin was used as a mature adipocyte marker and as expected its protein level was considerably increased at the late adipogenic stage (D12 post-differentiation) (Fig. 4A). During adipogenesis, changes in protein levels of ARL15 in the membrane-enriched fraction exhibit a different trend compared to those in the total cell lysate; whereas the Golgi marker RCAS1 did not show such difference (Fig. 4A). The level of ARL15 (relative to GAPDH) in the membrane-enriched fraction was significantly reduced at the late stage of adipogenesis (D12, post-differentiation) compared to earlier time points (Fig. 4B), suggesting that ARL15 dissociates from the membrane at a late stage of adipogenesis. Having observed changes in cellular localisation of ARL15 during adipocyte differentiation we wanted to further investigate the palmitoylation status of the protein during this process. Therefore, we performed an S-palmitoylation acyl-RAC assay over the time course of adipogenesis in 3T3L-1 cells. In agreement with the previous observation of ARL15 palmitoylation there was a trend towards reduction between post-adipogenic time point D4 and D12 (*P*=0.06) (Fig. 4C,D) although this was not statistically significant possibly due to variation between multiple repeats in this assay.

Since ARL15 is predominantly co-localised with the Golgi network membrane protein 58 kDa protein in preadipocytes, we next asked if ARL15 changes its sub-Golgi localisation during the adipocyte differentiation process in hWAT cells. Cells at pre-adipogenic and post-adipogenic stages (D0, D5, D10 and D15 post-differentiation induction) were subjected to immunostaining. We found that in hWAT preadipocytes (D0), endogenous ARL15 is co-localised with Golgi marker 58 kDa protein (Fig. 4E), consistent with previous findings. However, after the induction of adipogenesis, we found that the Golgi network changed from its canonical stack of tubular structures to a ring shape. ARL15 was then no longer co-localised with the 58 kDa protein and became confined within the Golgi ring. This localisation remained unchanged even 15 days post differentiation (Fig. 4E), suggesting that although predominantly localised in Golgi, ARL15 changes its sub-Golgi localisation during adipogenesis.

**Fig. 5.**
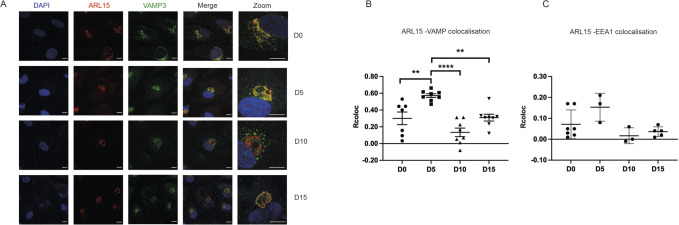
**ARL15 partially co-localises with VAMP3 at early stage of adipogenesis.** (A) Representative confocal images of hWAT cells co-stained with anti-ARL15 and anti-VAMP3 protein (recycling endosome localised protein). Secondary antibodies: Alexa-488 and Alexa-647 conjugated. Scale bars: 10 µm; Scale bars for zoomed-in image are 5 µm. Co-localisation analysis (Pearson correlation coefficient) during differentiation for ARL15 and VAMP3 (B) and ARL15 and EEA1 (C) proteins. Co-localisation coefficients are presented as mean±s.e.m. and analysed by using one-way ANOVA. **P<0.01, ****P<0.0001.

As the recycling endosome is also spatially confined within the Golgi ring (Misaki et al., 2007), we tested whether ARL15 is also localised in the recycling endosome and trafficked away from Golgi. We co-stained hWAT cells at pre-adipogenic and post-adipogenic stages (D0, D5, D10 and D15 post-differentiation induction) with ARL15 and the recycling endosome marker VAMP3. We found that VAMP3 was distributed in both Golgi area and in the cytoplasm (Fig. 5A). The cytoplasmic VAMP3 exhibits small punctate morphology, likely to reflect the previous finding that VAMP3 is localised in both recycling and early endosome (Galli et al., 1994; McMahon et al., 1993). Pearson's correlation coefficients (PCC) were used to assess the degree of co-localisation between ARL15 and VAMP3. PCC was significantly higher at D5 than other time points (Fig. 5B), suggesting that ARL15 transiently co-localises with VAMP3 during early stages of adipogenesis. In addition, the co-localisation was only visible within the Golgi area, not in small punctate cytoplasmic-distributed structures (Fig. 5A), suggesting that ARL15 stays in Golgi network area during adipogenesis.

To exclude the possibility of ARL15's localisation in early endosome, hWAT cells at D0, D5, D10 and D15 post-differentiation induction were co-stained with ARL15 and an early endosome marker EEA1 (Fig. S3). However, co-localisation was not seen at any time points between D0 and D15 as evaluated by Pearson's coefficients, which were all below 0.25, suggesting the two proteins are unlikely to co-localise and that ARL15 does not localise in early endosome (Fig. 5C).

### *Arl15* overexpression reduces levels of late-stage adipogenesis-related genes

**Fig. 6.**
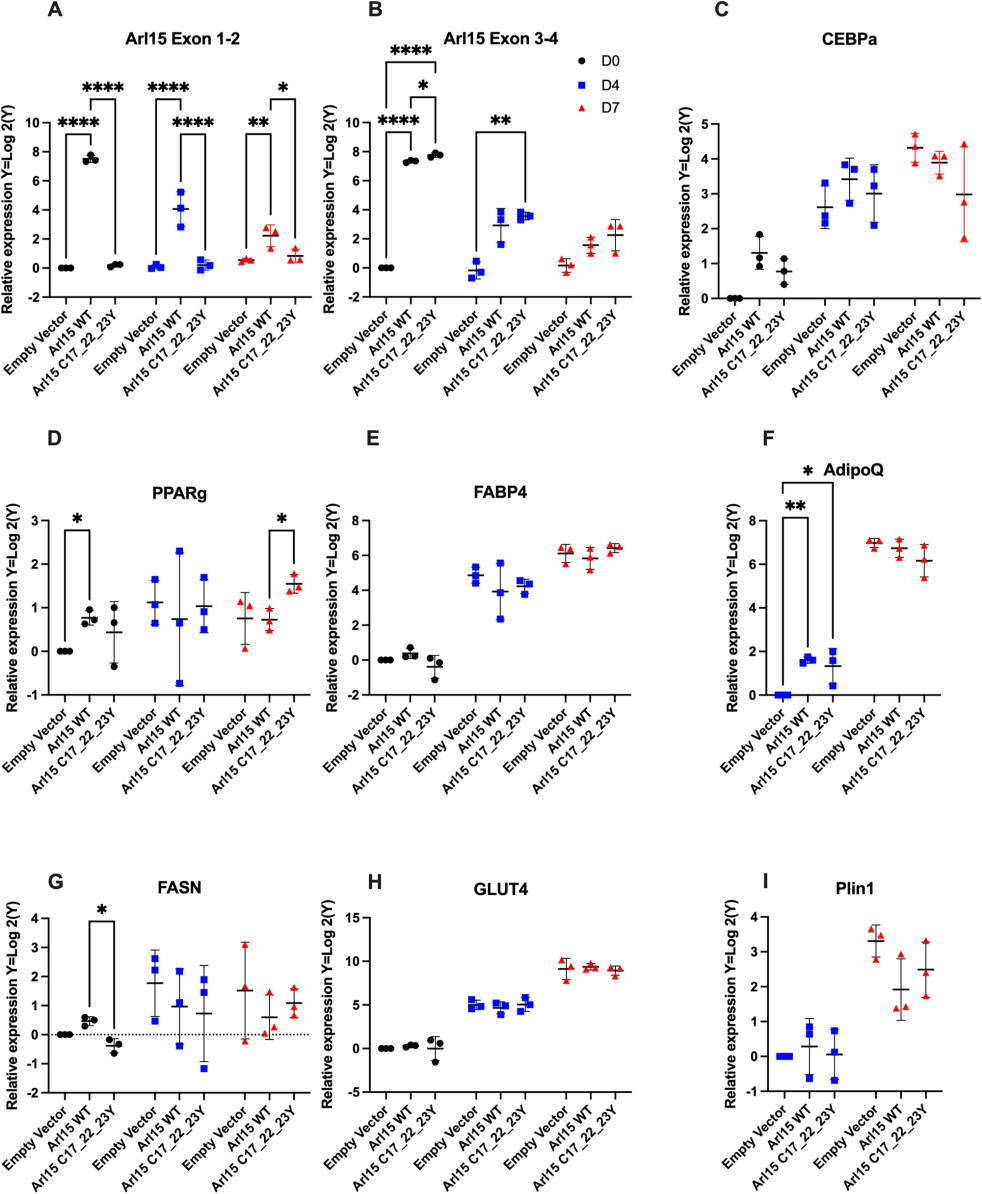
***Arl15* overexpression reduces levels of late-stage adipogenesis-related genes.** (A–I) q-RT-PCR for relative expression of *Arl15*, adipogenic and lipogenic genes in cells over-expressing WT and mutant ARL15 17_22_23Y in 3T3L1 cells at day 0, 4 and 7 of differentiation. Data are normalised to empty vector at day 0, with the exception of *AdipoQ* and *Plin1* that are expressed at very low levels at that time point and are consequently normalised to day 4. Data are Log 2 transformed with mean±s.d.from triplicates and analysed using two-way ANOVA with comparisons between the three vectors at each timepoint using Tukey's multiple comparison test. *P<0.05; **P<0.01; ****P<0.0001.

To study potential effects of *Arl15* overexpression in adipogenesis and roles that ARL15 palmitoylation may play on adipocyte differentiation, C-terminal Myc tagged empty vector control, *Arl15* WT or mutant (C17Y/C22Y/C23Y) was expressed from a human cytomegalovirus (CMV) promoter in 3T3-L1 cells prior to adipogenesis and gene expression analysis carried out on cells over a differentiation time-course at D0 (2 days post transfection) and at D4 and D7 post differentiation induction (Fig. S4; Fig. 6A–I). The levels of *Arl15* over-expression were assessed using two different TaqMan probes targeting exons 1-2 and 3-4 of WT *Arl15* (NCBI Reference Sequence: NM_172595.4), respectively. As expected, qPCR confirmed a significant over-expression of *Arl15* WT by both TaqMan probes at D0 compared to the empty vector control (Fig. 6A,B), whereas in the *Arl15* mutant group, the probe that only covers the non-mutation-containing exon 3 and 4 can detect a similar level of over-expression (Fig. 6B), demonstrating successful expression of both WT and mutant *Arl15*. The master adipogenesis regulator *Ppar*γ showed upregulation in *Arl15* WT in comparison to empty vector at D0 and upregulation in mutant compared to *Arl15* WT expressing cells at day 7 (Fig. 6D). The mature adipocyte marker adiponectin (*AdipoQ*) showed some upregulation at D4 comparing both WT and mutant overexpressing cells to empty vector but not at the later time point (Fig. 6F). The fatty acid synthase (*Fasn*) gene showed some evidence of reduction in mutant cells compared to WT at D0 but not at other timepoints (Fig. 6G). The early adipogenesis-related gene CCAAT enhancer binding protein alpha (*Cebpα*) and markers of differentiation, Fatty Acid Binding Protein 4 (*Fabp4*), glucose transporter type 4 (*Glut4*) and perilipin1 (*Plin1*) showed no differences although there may be a trend (*P*=0.0664) for reduction of *Plin* 1 in WT compared to empty vector (Fig. 6C,E,H,I).

Cytoplasmic lipid droplet levels were also tested using Oil Red O staining at D7. There were no significant differences detectable in either *Arl15* WT or mutant group, comparing to the empty vector control (data not shown). Taken together, these results show that *Arl15* WT or mutant over-expression only impaired adipogenesis to a limited extent.

### ARL15 interacts with ARL6IP5

**Fig. 7.**
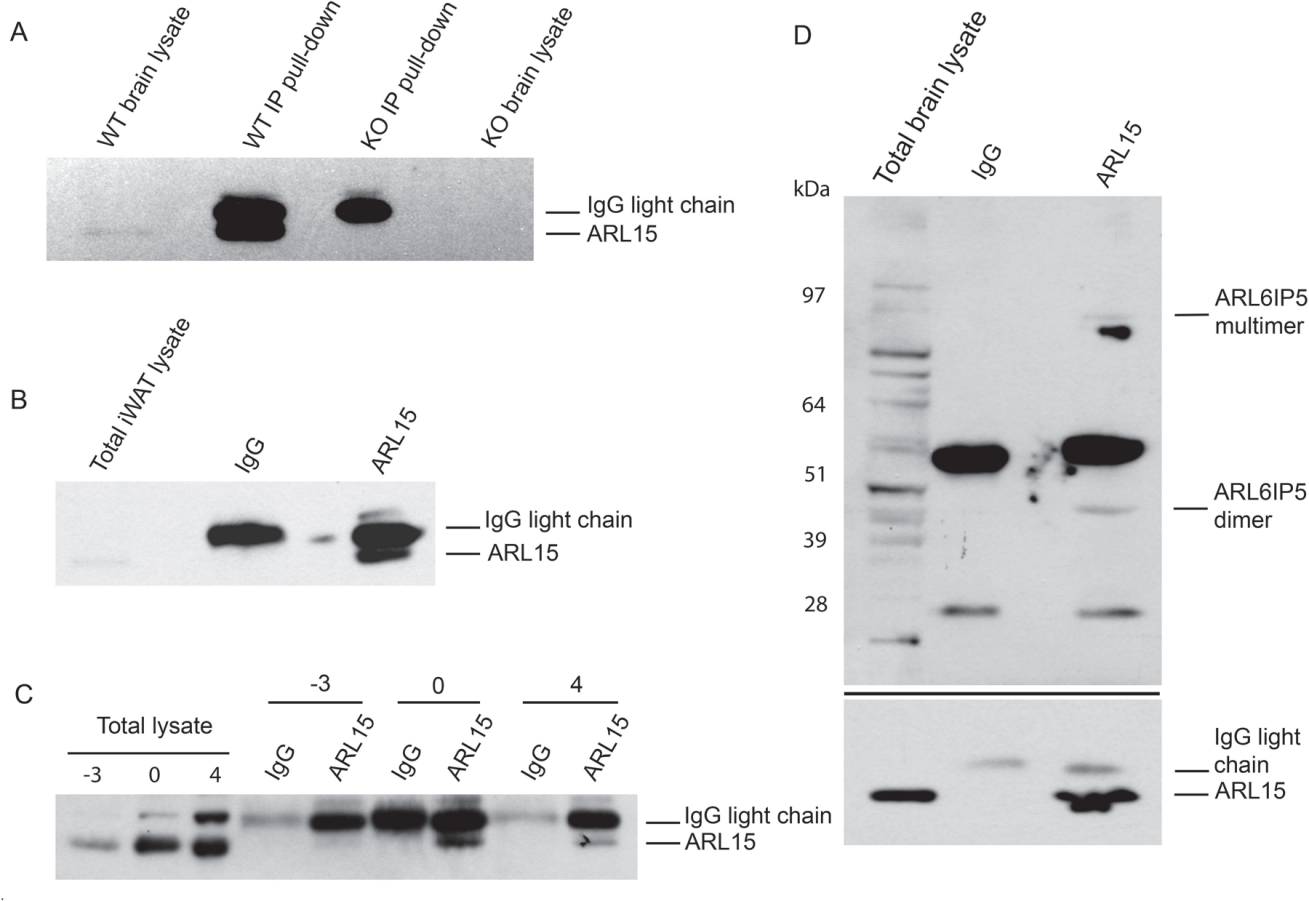
**Protein**–**protein interactions between ARL15 and ARL6IP5.** (A) Mouse brain lysates and those from homozygous *Arl15* knockout mouse were immunoprecipitated with an ARL15 antibody and probed for the presence of ARL15 by western blot analysis. Total lysates were also probed for the presence of the endogenous ARL15. (B) The mouse iWAT lysates were immunoprecipitated with an ARL15 antibody and probed for the presence of ARL15 by western blot analysis. As a control, the lysates were also incubated with a rabbit IgG antibody. Total lysates were probed for the presence of the endogenous ARL15. (C) The 3T3-L1lysates were immunoprecipitated with an ARL15 antibody and probed for the presence of ARL15 by western blot analysis. As a control, the lysates were also incubated with a rabbit IgG antibody. Total lysates were probed for the presence of the endogenous ARL15. (D) Mouse brain lysate was immunoprecipitated with an ARL15 antibody and probed for the presence of ARL15 (or ARL6IP5) by western blot analysis. As a control, the lysates were also incubated with a rabbit IgG antibody. Total lysates were probed for the presence of the endogenous ARL15 and ARL6IP5.

The Golgi network is the hub of vesicle trafficking and protein–protein interactions. To identify interacting partners of ARL15, immunoprecipitation (IP) was carried out using an anti-ARL15 antibody to pull down ARL15 from brain lysate from WT and homozygous KO mice (Fig. 7A), WT subcutaneous adipose tissue lysate (Fig. 7B) and 3T3-L1 cells at different timepoints during adipogenesis (D-3, D0 and D4 post differentiation induction) (Fig. 7C). Western blotting demonstrated successful pull-down of ARL15, as the ARL15 protein band was only detectable in the pull-down lanes, not in control IgG or knockout mouse tissue lanes (Fig. 7A–C). Potential protein–protein interacting complexes from whole immune-precipitation samples were then analysed by mass spectrometry. Several interacting partners were identified (Table 1). Among them, ADP Ribosylation Factor like GTPase 6 Interacting Protein 5 (ARL6IP5) also named as JWA and GTRAP3-18 was identified as a potential interacting partner of ARL15 in all samples. Importantly, for mouse brain lysate, homozygous *Arl15* knockout mouse brain was used as a negative control for the western blot and mass spectrometry analysis instead of IgG, excluding the possibility of antibody non-specificity. ARL6IP5 was detectable by mass spectrometry only in WT mouse brain lysate pulled down by anti-ARL15 antibody, not in the homozygous *Arl15* knockout control (Table 1), further suggesting that ARL6IP5 is a potential interacting partner of ARL15. To confirm the interaction between ARL15 and ARL6IP5, WT mouse brain lysate was subjected to immunoprecipitation followed by western blotting. ARL6IP5 bands were detectable in ARL15 pulled-down samples, but not in the IgG control, demonstrating the interaction between ARL15 and ARL6IP5 (Fig. 7D). Of note, only dimer and multimer forms of ARL6IP5 were detectable, consistent with previous findings that ARL6IP5 also exists as non-monomeric forms in the brain and other cell types (Lin et al., 2001; Vento et al., 2010).

**Table 1.**
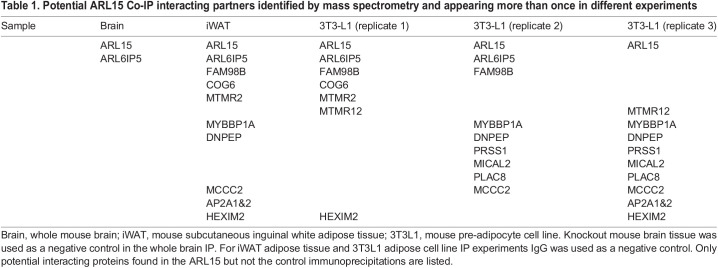
Potential ARL15 Co-IP interacting partners identified by mass spectrometry and appearing more than once in different experiments

## DISCUSSION

In the present study, results from immunocytochemistry demonstrated that both endogenous ARL15 in hWAT preadipocytes and overexpressed WT ARL15 in 3T3-L1 preadipocytes co-localised with the Golgi network and in co-localising with 58 kDa Golgi protein likely faced the cytosol (Bloom and Brashear, 1989).

Previously, Ren et al. conducted proteomic analysis and showed that two ARL family proteins, ARL3 and ARL15, were palmitoylated in adipose tissues (Ren et al., 2013). Using stable isotope labelling by amino acids in cell culture (SILAC), ARL15 was also found to be palmitoylated in human umbilical vein endothelial cells (HUVECs) (Wei et al., 2014). Using the acyl-RAC assay, we have confirmed palmitoylation of ARL15 in mouse adipose tissue and Golgi isolated from mouse liver. Numerous peripheral membrane proteins have been found to be palmitoylated at the surface of Golgi (Rocks et al., 2010), while palmitoylated integral membrane cargo proteins are found to be in the rims of cis-Golgi (Ernst et al., 2018), suggesting that sub-Golgi localisation may be important to a protein's function. Since the hydrophobic palmitoyl group enables a reversible protein-membrane association this may facilitate reversible association of ARL15 with Golgi membranes (Charollais and Van Der Goot, 2009; McLaughlin and Denny, 1999). Indeed, we found that in hWAT cells, ARL15 was translocated away from Golgi surface, suggesting a potential depalmitoylation process after adipogenic induction, consistent with a role for ARL15 in protein and membrane trafficking in adipocytes. Interestingly, a number of proteins involved in lipid formation and metabolism are also palmitoylated (Ren et al., 2013). ARL15 has been reported to be translocated from the cytoplasm to Golgi around the nucleus on insulin stimulation of muscle C2C12 cells (Zhao et al., 2017), however, our immunostaining data demonstrated that in the absence of insulin stimulation, both endogenous ARL15 in hWAT preadipocytes and over-expressed ARL15 in 3T3-L1 preadipocytes were also established at a perinuclear Golgi localisation. These may be cell specific differences.

To date, the significance of ARL15 palmitoylation in adipogenesis remains to be elucidated. In our study, 3T3-L1 cells over-expressing WT and palmitoylation mutant *Arl15* (C17_22_23) were differentiated and there was some evidence of upregulation of the adipogenesis master regulator *Pparγ* at early and late timepoints and an upregulation of the mature differentiation *AdipoQ* at day 4 in both WT and mutant cells. Conversely there was a reduction in the differentiation marker *FASN* in mutant cells, although this was only at an early timepoint where expression is expected to be low. However, it should be noted that not all cells are likely to be overexpressing ARL15 in these transfection experiments. A more direct test of the importance of palmitoylation in adipogenesis would be possible using engineered cells expressing endogenous levels of ARL15.

Links between palmitoylation of other ARL family proteins and adipogenic regulation have been observed. For instance, ARL13B has palmitoylation-dependent roles on cilia formation and Sonic hedgehog (Shh) pathway regulation (Larkins et al., 2011; Roy et al., 2017). Shh protein treatment on 3T3-L1 and NIH-3T3 cells inhibited adipogenesis, demonstrating a negative regulatory role of Shh pathway on adipogenesis (Suh et al., 2006). Additionally, cilia carrying hedgehog pathway proteins transiently appeared in human white preadipocyte (HWP) during adipogenesis, whereas cilia deformation resulted in upregulation of adipogenic master regulator PPARγ (Bangs and Anderson, 2017; Marion et al., 2009).

Two important adipose proteins, the hormone adiponectin and the glucose transporter GLUT4, have also been shown to be palmitoylated in adipose tissue (Ren et al., 2013). Overexpression of a palmitoylation mutant (Cys223) of GLUT4 resulted in change of subcellular localisation and impairment of insulin dependent membrane translocation (Ren et al., 2015), suggesting an important role for palmitoylation in facilitating membrane trafficking of GLUT4. However, ARL15 does not appear to be directly involved in trafficking of adiponectin or GLUT4, as adiponectin was not co-localised with ARL15 as reported by Rocha et al. (Rocha et al., 2017) and in our co-immunoprecipitation experiments we also did not find evidence of direct interaction between adiponectin or GLUT4 proteins in mouse iWAT. Further, 5 days after induction of adipogenesis, we could not immunocytochemically detect co-localisation between ARL15 and GLUT4 in hWAT cells incubated in insulin-containing medium. Similarly, Rocha et al. found that knocking down *Arl15* had little impact on GLUT4 translocation-mediated glucose uptake (Rocha et al., 2017).

Other Golgi localised ARL family proteins, for example, ARL1, ARL5 and ARL18 (ARFRP1) are all mediators of vesicle/membrane trafficking and transport (Houghton et al., 2012; Lowe et al., 1996; Shin et al., 2005) and is a plausible role for ARL15. However, the cargo specificity and relationship between the Golgi localisation of palmitoylated ARL15 and intracellular trafficking needs to be further studied.

A novel protein–protein interacting partner of ARL15 has been reported. Using Co-IP followed by mass spectrometry, a pioneer study revealed that the ArfGAP protein containing a SH3 Domain, Ankyrin Repeat And PH Domain 2 (ASAP2) interacts with ARL15 (Zhao et al., 2017). However, ASAP2 was identified from C2C12 skeletal muscle cell samples where insulin-mediated translocation of ARL15 to Golgi was detected, whereas in our 3T3-L1 or hWAT samples, such translocation was not seen, suggesting potentially different interacting partners. Indeed, we did not identify ASAP2 by mass spectrometry from Co-IP 3T3-L1 samples at different adipogenic timepoints or from mouse iWAT lysate. We identified ARL6IP5 as an interacting partner of ARL15 not only in adipose-related cell types and tissue, but was also in brain samples, indicating common pathways that ARL15 may be involved in brain and adipose tissue. Predominantly localised in ER (Maier et al., 2009), ARL6IP5 has been found to reduce ER-Golgi trafficking by interacting with ER-Golgi transport regulator RAB1 and such interaction exhibited an inhibitory effect on neuronal differentiation (Maier et al., 2009). Since our results revealed that ARL15 is likely to be localised at the ER-facing cis Golgi network, such localisation indicates that ARL15 may be involved in mediating ER-Golgi transport via interacting with ARL6IP5. In addition to the potential role of ARL6IP5 in regulating neuronal differentiation, knocking down *Arl6ip5* in UAMS-32 osteoblastic cell line has been reported to reduce cell proliferation and differentiation (Wu et al., 2014). Such a role is consistent with a regulatory role in adipocyte differentiation (Rocha et al., 2017).

In conclusion, we demonstrated that endogenous ARL15 is palmitoylated in subcutaneous adipose tissue and palmitoylation seems to be involved in maintaining the Golgi localisation of ARL15. ARL15 interacts with ARL6IP5 an ER-localised protein and translocates within the Golgi network during adipogenic differentiation consistent with a role in protein trafficking. Taken together, these results indicated a palmitoylation dependent Golgi-based role of ARL15 as a regulator of cell differentiation.

## MATERIALS AND METHODS

### Primers and cloning

Primers used for the site-directed mutagenesis were synthesised by Sigma-Aldrich and pCMV-Arl15-Myc-DDK vector was used as a template. Primer sequences are: C17Y: forward: 5′-ATGGATTATCTATGTTTTCGAGCG-3′, and a reverse: 5′-GTACAGAAACGCCTCAGTTATC-3′; C22Y: forward: -5′-GCTTTACTGCAAGGGACCAC-3′, and a reverse: 5′- GCTCGAAAACACAGATAATCC-3′ ; C23Y: forward: −5′-GCTTTGCTACAAGGGACCAC-3′ and reverse: 5′- GCTCGAAAACACAGATAATCC-3′ ;C22_23Y: forward: 5′- GCTTTACTACAAGGGACCAC-3′, and reverse: 5′- GCTCGAAAACACAGATAATCC-3′. Site-directed mutagenesis and Kinase, Ligase & DpnI (KLD) treatment were conducted using Q5^®^ Site-Directed Mutagenesis Kit as per the manufacturer's instructions. Transformation was carried out by mixing a 50 μl of NEB 5-α Competent *E. coli* with 5 μl of the KLD product and placed on ice for 30 min. Then, the mixture was heat shocked at 42°C for 30 s followed by placing on ice for 5 min. 950 μl of room temperature SOC was pipetted into the mixture followed by a 60-min incubation at 37°C, 250 rpm. Then, 50–100 μl of the transformation mix in SOC was plated onto a kanamycin selection plate and incubated overnight at 37°C. Constructs were verified by Sanger sequencing.

### Cell culture

3T3-L1 (ATCC, CL-173) or human white adipocyte (hWAT, provided by Prof. Yu-Hua Tseng, PhD at Harvard Medical School, Joslin Diabetes Center, Boston, MA 02215, USA; Xue et al., 2015) cells were maintained in Dulbecco's Modified Eagle's Medium (DMEM) with glucose, containing L-glutamine and supplemented with 10% NBCS or FBS and 1% Penicillin-streptomycin mixture (Life Technologies) at 37°C, 5% CO2. Cells were checked for mycoplasma and were negative. Cells at different passage were used as replicates. To induce adipogenesis in 3T3-L1 preadipocytes, differentiation medium containing DMEM (Thermo Fisher Scientific, CAT. No. 31966021) supplemented with 10% FBS, 1% Penicillin-streptomycin mixture (Life Technologies), 500 µmol/l IBMX, 1 µmol/l dexamethasone, 10 µmol/l insulin was added to 2-day post-confluent 3T3-L1 preadipocytes and cultured for 4 days. Cells were further cultured in maintenance medium containing DMEM supplemented with 10% Tet-Approved FBS, 10 µmol/l insulin (Merck, CAT. No. I9278) until harvest for analysis. Adipogenesis induction in hWAT cells was adapted from Xue et al. with modifications (Xue et al., 2011). In brief, differentiation medium containing DMEM (Thermo Fisher Scientific) supplemented with 2% FBS, 1% Penicillin-streptomycin mixture (Life Technologies), 2 nmol/l T3, 33 µmol/l biotin, 17 µmol/l pantothenate, 30 µmol/l indomethacin, 0.5 mmol/l IBMX, 0.1 µmol/l dexamethasone and 0.5 µmol/l insulin was added to post-confluent hWAT preadipocytes and cultured until harvest for analysis.

### Golgi network isolation

Golgi isolation was carried out by using a Golgi isolation kit (Sigma-Aldrich) as per the manufacturer’s instructions with minor modifications. Mouse liver tissue was first washed with ice-cold PBS, then washed once with 10 ml of 0.25 M sucrose solution and weighed. The tissue was then minced with scissors. The minced tissue was then suspended with 1 ml of the 0.25 M sucrose solution per 1 g of tissue. Tissue suspension was then transferred into a homogenisation tube and homogenised with six slow motions of the PTFE pestle at 300 rpm. The homogenate was then centrifuged at 3000×***g*** for 15 min at 2–8°C. After centrifugation, the supernatant was transferred to a fresh tube. The sucrose concentration in the supernatant was calculated according to Eqn (1): [Sucrose]=0.25*Buffer Volume/ (Tissue Weight+Buffer Volume). Where: [Sucrose] is the molar sucrose concentration in the supernatant; 0.25 is the molar sucrose concentration in the homogenization buffer; Buffer Volume is the volume (ml) of the buffer used to re-suspend tissue after mincing; Tissue Weight is the weight of tissue in grams. The volume of the 2.3 M sucrose solution to be added to the supernatant to obtain a final sucrose concentration of 1.25 M was calculated according to Eqn (2): Sucrose Volume=Sup Volume *(1.25−[Sucrose])/1.05. Where: Sucrose Volume is the volume (ml) of 2.3 M Sucrose Solution to be added to the supernatant in order to obtain a final concentration of 1.25 M sucrose; Sup Volume is the volume (ml) of the supernatant; [Sucrose] is the molar sucrose concentration in the supernatant from Equation (1). 1.05 is the difference in the molarity between the sucrose stock solution (2.3 M) and the sucrose concentration of the sample (1.25 M).

After calculation, the sucrose concentration in the sample (supernatant) was adjusted to 1.25 M by adding the volume of 2.3 M sucrose solution calculated in Eqn (2). The solution was mixed by inverting the tube followed by a brief vortex. A discontinuous gradient in an ultracentrifuge tube was then made. The order of sucrose gradient fractions in the tube (from bottom to top) were: 1.84 M sucrose solution, sample (sucrose concentration adjusted to 1.25 M), and 1.1 M sucrose solution and 0.25 M sucrose. The tubes were then centrifuged at 120,000×***g*** for 3 h at 4°C. The Golgi enriched fraction was at the 1.1 M/0.25 M sucrose interphase.

### Protein extraction, antibodies and western blotting

3T3-L1 cells were lysed in M-PER™ Mammalian Protein Extraction Reagent (Thermo Fisher Scientific), mouse tissues were lysed in T-PER Tissue Protein Extraction Reagent (Thermo Fisher Scientific) as per the manufacturer's instructions, supplemented with protease and phosphatase inhibitors (Roche). Protein concentrations of lysates were measured against a serial dilution of BSA standards using the *DC™* (detergent compatible) protein assay (Bio-Rad). 10 µl of 4X LDS Sample loading buffer (Life Technologies) and 4 µl of 10X reducing agent (Life Technologies) were added to 20 μg of protein lysates, heat-denatured at 95°C for 5 min and then loaded on 4–12% Bis-Tris NuPAGE precast gels (Life Technologies). Proteins were transferred to 0.45 µm PVDF membranes (GE Healthcare) using the XCell II™ Blot module (Life Technologies). Membranes were first incubated in blocking solution [5% dried non-fat milk prepared in Tris-buffered saline containing 0.1% Tween-20 (TBST)] at room temperature for an hour on a shaking platform, washed with TBST and then incubated with primary antibodies overnight at 4°C on a shaking platform. The membranes were washed three times with TBST and finally incubated with HRP-conjugated anti-rabbit or anti-mouse (Bio-Rad), secondary antibody prepared in blocking solution for 1 h at room temperature. After washing excess antibody, the Pierce™ ECL Plus Western Blotting Substrate was used for signal detection using the UVP ChemiDoc-It™ Imaging System.

The following antibodies were used: rabbit anti-ARL15 (Abcam ab178425, 1:1000), mouse anti-GAPDH (Abcam ab8245, 1:10,000), mouse anti-adiponectin (Abcam ab22554, 1:1000), mouse anti-ARL13B (Abcam ab136648, 1:200), mouse anti-RCAS1 (Cell Signaling Technology #678565, 1:1000) and rabbit anti-ARL6IP5 (Sigma-Aldrich SAB1306837, 1:1000).

### Quantitative real time PCR (qPCR)

RNA was extracted from 3T3-L1 cells using Direct-zol RNA Kit (Zymo Research) as per the manufacturer's instructions and the concentration measured using a Nanodrop system. cDNA was generated using Superscript III reverse transcriptase enzyme (Life Technologies) and analysed by qPCR using the TaqMan system based on real-time detection of accumulated fluorescence (ABI Prism 7700, Perkin-Elmer Inc., USA). Samples were tested in triplicate and gene expression normalised relative to the expression of house-keeping genes *CanX* (Mm00500330_m1) and *Rpl13a* (Mm01612986_gH). *Arl15* (Mm01304728_m1 Exon1-2 and Mm00553694_m1 Exon 3-4), *C/ebpα* (Mm00514283_s1), *C/ebpβ* (Mm00843434_s1), *Pparγ* (Mm01184322_m1, *Fabp4* (Mm00445878_m1), *Glut4* (Mm00436615_m1), *AdipoQ* (Mm00456425_m1), *Plin1* (Mm00558672_m1) and *Fasn* (Mm00662319_m1) FAM labelled TaqMan Probes were purchased from Applied Biosystems (ABI, USA).

### Immunofluorescence

Cells were grown on coverslips in 12-well plates. After washing with PBS, cells were fixed using 4% PFA and permeabilised using 0.1% Triton X-100 (Sigma-Aldrich). To block non-specific antibody binding, cells were blocked for 1 h at room temperature in blocking buffer containing 5% donkey serum, Tween-20 (Bio-Rad) and Glycine (Sigma-Aldrich). Staining was carried out using primary antibodies with optimised concentrations overnight at 4°C followed by three washes with PBST. Alexa fluorophore conjugated secondary antibodies were used to detect primary antibody-labelled proteins for 1 h at room temperature. DNA stain NucBlue present in mounting medium (Thermo Fisher Scientific, P36985) or DAPI was used to stain cell nuclei. DAPI stained cells were mounted onto slides using the DAKO fluorescent mounting medium. Cells were imaged on the Zeiss inverted confocal microscope (Carl Zeiss, Switzerland)

Primary antibodies used: rabbit anti-ARL15 (Sigma-Aldrich HPA063820, 1:100), rabbit anti-tagged Myc antibody (Abcam ab9106, 1:500), mouse anti-58 kDa protein (Abcam ab27043, 1:100), mouse anti-VAMP3 antibody (Santa Cruz Biotechnology sc-514843, 1:100), mouse anti-EEA1 antibody (Abcam ab70521, 1:100), mouse anti-Myc (Invitrogen 132500, 1:200), rabbit anti-calreticulin (Abcam ab92516, 1:500). The following secondary antibodies were used: anti-mouse Alexa-568 (Thermo Fisher Scientific A10037, 1:500) and anti-rabbit Alexa-488 (Thermo Fisher Scientific A32790, 1:500) or goat anti-rabbit Alexa 488 (Abcam ab150077, 1:1000) and goat a-mouse-Alexa 647 (Abcam ab150115, 1:250).

### Cell membrane enrichment

The cell membrane enrichment method was adapted from (Forrester et al., 2011) with minor modification. In brief, 3T3-L1 cells were collected and washed in cold PBS. After undergoing a freeze-thaw cycle, cells were resuspended in lysis buffer (25 mM HEPES, 25 mM NaCl, 1 mM EDTA, pH 7.5) containing protease inhibitor cocktail (Roche). Lysis was conducted by repeated passaging through a 28-gauge needle. Then cell lysates were centrifuged at 800 ***g*** for 5 min to get rid of nuclei and unbroken cells. The supernatant was then centrifuged at 20,000 ***g*** for 30 min at 4°C. The pellet was re-suspended in lysis buffer containing 0.5% Triton X-100. Total protein was quantified with a DC™ protein assay (Bio-Rad).

### Acyl-RAC assay

Methodology for acyl-RAC, including blocking of free thiols with methyl methanethiosulfonate (MMTS) (Sigma-Aldrich 208795-1G, Catalog number: 23011, pH 7.5), cleavage of thioester linkages, and capture of nascent thiols on thiopropyl sepharose, was from (Forrester et al., 2011) with minor modifications. In brief, equal amounts of protein (2.0 mg) were diluted to a concentration of 2 mg/ml in (total volume 1 ml) blocking buffer (100 mM HEPES, 1.0 mM EDTA, 2.5% SDS, 0.1% MMTS, pH 7.5) and incubated at 40°C for 10 min with frequent vortexing. Three volumes of cold acetone were then added, and proteins were subjected to precipitation at −20°C for 20 min. Then the solution was concentrated at 5000 ***g*** for 10 min, the pellet was extensively washed with 70% acetone, re-suspended in 300 μl of binding buffer (100 mM HEPES, 1.0 mM EDTA, 1% SDS, pH 7.5) and added to ∼40 μl of prewashed thiopropyl sepharose beads (GE Amersham). To this mixture was added 40 μl of either 2 M NH2OH (freshly prepared in H2O from HCl salt and brought to pH 7.5 with concentrated NaOH) or 2 M NaCl. Binding reactions were carried out on a rotator at room temperature for 2–4 h. Approximately 20 μl of each supernatant was saved as the ‘total input’. Resins were washed at least five times with binding buffer. For immunoblot analysis, elution was performed using 60 μl of binding buffer containing 50 mM DTT at room temperature for 20 min. Supernatants were removed and mixed with Laemmli loading buffer, heated to 95°C for 5 min, and separated via SDS-PAGE on a Mini-Gel apparatus (Bio-Rad). For Figs 3, 4 and Fig. S2, pull down of S-palmitoylated proteins was performed with the commercial kit CAPTUREome™ S-Palmitoylated Protein Kit (Badrilla, K010-311). Briefly proteins were extracted in 1X Binding Buffer A and were quantified by Pierce BCA protein kit assay (Thermo Fisher Scientific). All proteins were then diluted to the same concentration. 50 µl of each sample was then kept as the input fraction and the rest of the proteins were subjected to the resin-assisted capture assay according to the manufacturer's protocol.

### Immunoprecipitation

3T3-L1 cells were lysed in M-PER™ Mammalian Protein Extraction Reagent (Thermo Fisher Scientific), mouse tissues were lysed in T-PER Tissue Protein Extraction Reagent (Thermo Fisher Scientific) as per the manufacturer's instructions, supplemented with protease and phosphatase inhibitors (Roche) and 5 mM MgCl_2_ (Sigma-Aldrich). Protein G Sepharose beads (Sigma-Aldrich) were washed by 1 ml lysis buffer and centrifuged at 1000 ***g*** for 1 min at 4°C. Cell or tissue lysates with the pre-washed beads were then incubated for 1 h at 4°C followed by centrifugation at 1000 ***g*** for 1 min at 4°C to remove the beads. Then antibody was added to the sample lysate. The antibody-containing pre-cleaned lysate was incubated on a tube rotator overnight at 4°C. After overnight incubation, freshly pre-washed Protein G Sepharose beads (Sigma-Aldrich) were added to the antibody-containing pre-cleaned lysate and incubated for 1 h at 4°C. Then, beads were collected by centrifugation at 1000 ***g*** for 1 min at 4°C followed by washing the immune complexes six times using PBS supplemented with protease and phosphatase inhibitors to remove nonspecific binding. The protein–protein interacting partners were then eluted by boiling at 95°C for 5 min. Samples were then directly analysed by SDS-PAGE or stored at −20°C for subsequent use.

### Mouse tissue samples

All animal studies were approved by the Medical Research Council Harwell Institute Animal Welfare and Ethical Review Board, and all procedures were carried out within project license restrictions (PPL 30/2642 and 30/3146) under the UK Animals (Scientific Procedures) Act 1986 and following the ARRIVE guidelines for animal research. Mice were housed according to UK Home Office welfare guidelines in a 12 h light/dark cycle at a temperature of 21±2°C and humidity of 55±10%. Mice were fed *ad libitum* and had free access to water (25 ppm chlorine). Tissue samples were obtained from C57BL/6Ntac and Arl15-Del2-EM1-B6N mice. The Arl15-Del2-EM1-B6N mice carry a 2-nucleotide CRISPR/Cas9 induced deletion in exon 2 of the *Arl15* gene and was generated by the Molecular and Cellular Biology team at the MRC Harwell Institute.

### Statistical analysis

Statistical analysis was performed using the Graphpad Prism 8 software. Student's *t*-test, one- or two-way ANOVA with repeated measures with post-hoc Tukey's correction was applied. *P*-values ≤0.05 were considered significant. Data distribution normality was assessed by Shapiro-Wilk test (Graph Pad Prism 8). For equal variance assessment, *F*-tests were used and groups are with similar variance (*P*>0.05).

## Supplementary Material

Supplementary information
