# Supplementary material for: Palmitoylated small GTPase ARL15 is translocated within Golgi network during adipogenesis
Source: Biol Open. 2021 Dec 13;10(12):bio058420. doi: 10.1242/bio.058420 (PMC8689486; doi:10.1242/bio.058420)
Supplement: Supplementary information [file biolopen-10-058420-s1.pdf]

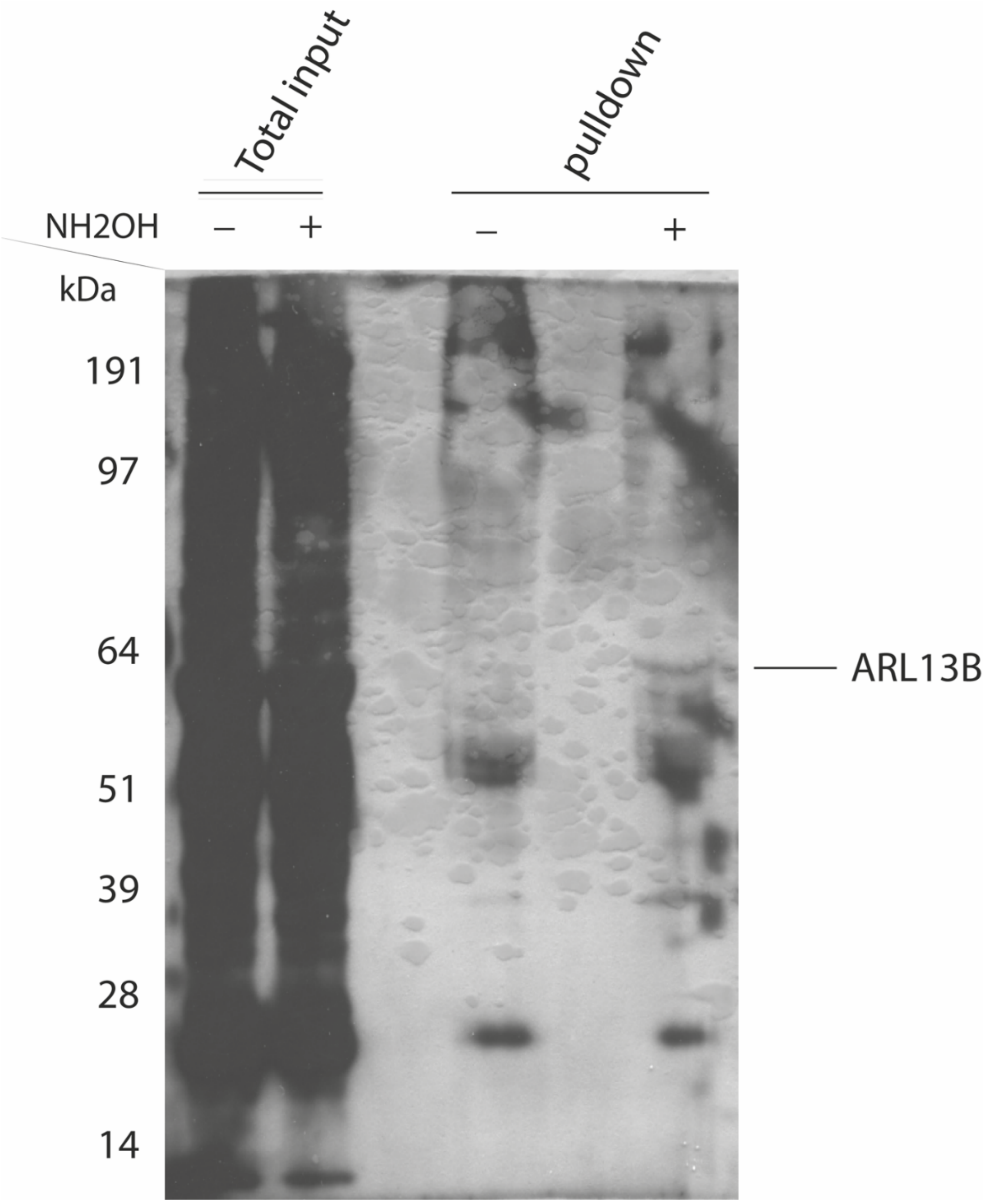

Fig. S1. ARL13B, a known palmitoylated protein is detectable in mouse inguinal white adipose tissue (iWAT) by RAC.

The detection of palmitoylation of ARL13b from mouse iWAT was carried out by acyl-RAC and western blot. Samples were treated with a final concentration of 0.2 M NH_2OH or 0.2 M NaCl as a control and then palmitoylated proteins were enriched using prewashed thiopropyl Sepharose beads. Enriched samples were eluted using binding buffer containing 50 mM DTT at room temperature for 20 minutes. Total input and eluted fraction from NaCl-containing control (indicated as "−") and NH_2OH-treated samples (indicated as "+") were separated by SDS-PAGE followed by western blotting with a primary antibody to ARL13B.

Biology Open • Supplementary information

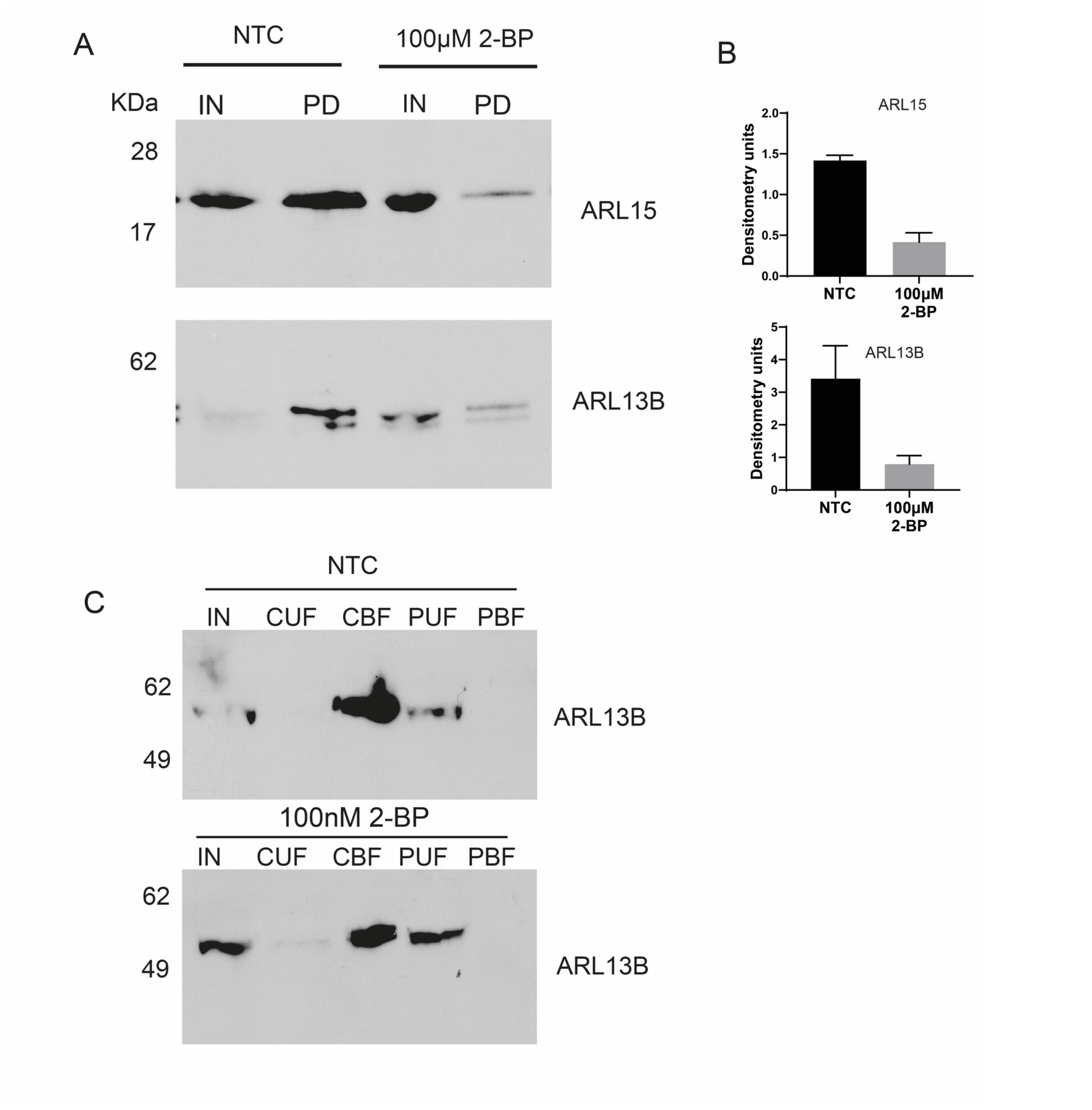

Fig. S2. 2-bromopalmitate (2-BP), a known blocker of palmitate incorporation inhibits ARL15 and ARL13B palmitoylation.

(A)Treatment with 100μM 2-BP overnight resulted in significantly reduced palmitoylation of ARL15 and ARL13B in 3T3-L1 cells compared to non-treated control cells (NTC) as detected by S-acylated resin-assisted capture (RAC) and western blot. (B) Densitometry for ARL15 and ARL13B. Densitometry is relative to input, data presented as mean±s.e.m, n=2. (C) Representative image of the S-acylated RAC generated fractions showing enrichment of the cleaved bound fraction, complete cysteine blocking and specific binding of protein in the resin as indicated by the absence of the preserved bound fraction. This example was also treated with 100nM 2-BP overnight, although this does not significantly inhibit palmitoylation. NTC: non-treated control lysates, PD: pull down lysates, IN: input fraction, CUF: cleaved unbound fraction, CBF: cleaved bound fraction/pull-down, PUF: preserved unbound fraction, PBF: preserved bound fraction.

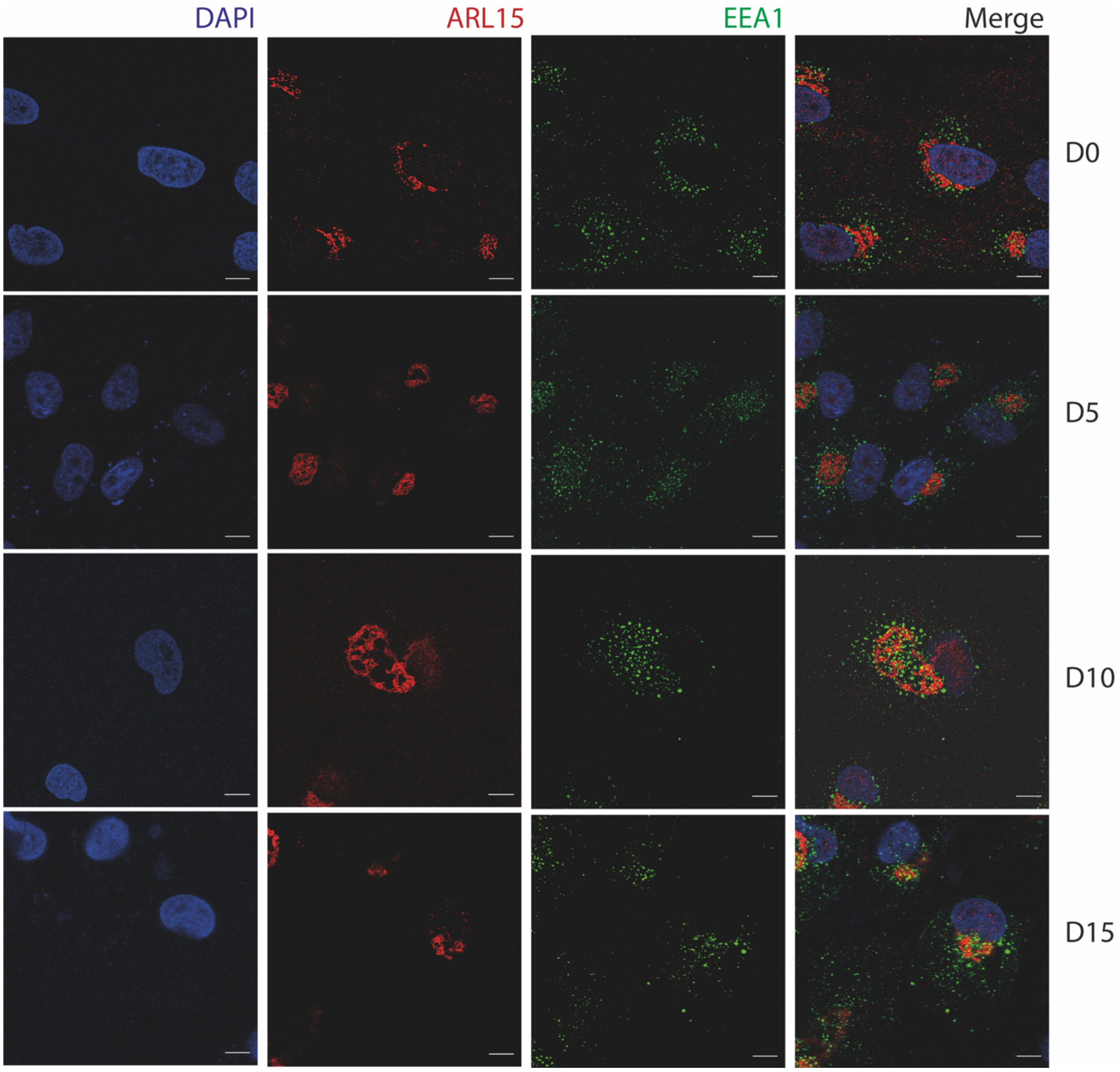

Fig. S3. ARL15 is not localised in early endosome.

Representative confocal images of hWAT cells were co-stained with anti-ARL15 and anti-EEA-1 (early endosome marker) antibodies followed by incubating with secondary antibody conjugated with Alexa-568 (red) and Alexa-488 (green). Scale bar=10 μm

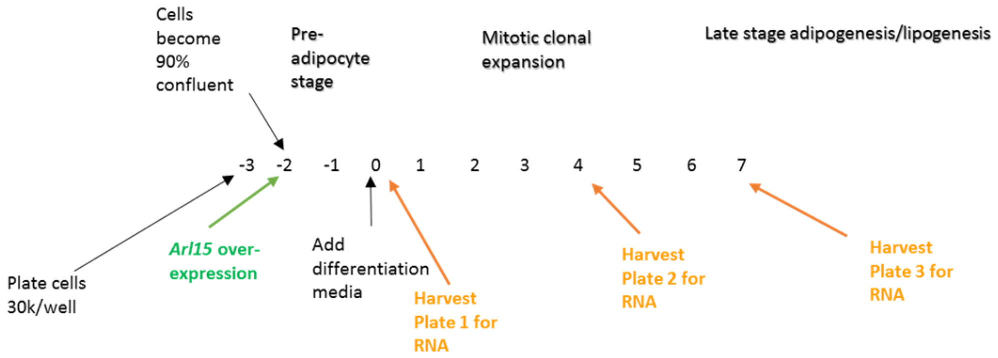

Fig. S4. Experimental plan for 3T3-L1 cell differentiation and Arl15 overexpression (WT and mutant Arl15 17_22_23Y) in 3T3-L1 adipocytes.

3T3-L1 preadipocytes were plated at 30k/well 3 days prior to adipogenesis induction (D-3). Cells were lipofectamine-transfected with WT or mutant Arl15 (C17_22_23Y) on D-2. 48 hours post-transfection, differentiation medium was added (D0). Cells were cultured until harvest for analysis at D0, D4 and D7 post-differentiation induction.

Biology Open • Supplementary information

Table S1. ARL15 has three palmitoylation sites at its N-terminal (Cys17, Cys22 and Cys23) predicted by the clustering and scoring strategy algorithm CSS-Palm 4.0. The score calculated by algorithm CSS-Palm corresponds to the confidence of the palmitoylation of the tested peptide; the cut-off score corresponds to the sensitivity and specificity of CSS-Palm (Zhou et al., 2006). A high threshold pre-set by the developer was selected for palmitoylation sites prediction.

ID	Position	Peptide	Score	Cutoff
sp|Q8BGR6|ARL15_MOUSE ADP-ribosylation factor-like protein 15 OS=Mus musculus OX=10090 GN=Arl15 PE=1 SV=1	17	FLYMDYLCFRALCCK	22.841	4.222
sp|Q8BGR6|ARL15_MOUSE ADP-ribosylation factor-like protein 15 OS=Mus musculus OX=10090 GN=Arl15 PE=1 SV=1	22	YLCFRALCCKGPPPA	7	3.419
sp|Q8BGR6|ARL15_MOUSE ADP-ribosylation factor-like protein 15 OS=Mus musculus OX=10090 GN=Arl15 PE=1 SV=1	23	LCFRALCCKGPPPAR	15.222	4.222

References

ZHOU, F., XUE, Y., YAO, X. & XU, Y. 2006. CSS-Palm: palmitoylation site prediction with a clustering and scoring strategy (CSS). *Bioinformatics,* 22**,** 894-896.

Biology Open • Supplementary information
